# Temporal GeneTerrain: advancing precision medicine through dynamic gene expression visualization

**DOI:** 10.3389/fbinf.2025.1602850

**Published:** 2025-06-18

**Authors:** Ehsan Saghapour, Rahul Sharma, Delower Hossain, Kevin Song, Zhandos Sembay, Jake Y. Chen

**Affiliations:** ^1^ Department of Biomedical Informatics and Data Science, The University of Alabama at Birmingham, Birmingham, AL, United States; ^2^ Systems Pharmacology AI Research Center, The University of Alabama at Birmingham, Birmingham, AL, United States; ^3^ Department of Computer Science, The University of Alabama at Birmingham, Birmingham, AL, United States; ^4^ Department of Biomedical Engineering, The University of Alabama at Birmingham, Birmingham, AL, United States

**Keywords:** cancer cell lines, gene expression, drug screening, precision medicine, bioinformatics, data visualization

## Abstract

**Introduction:**

Understanding the temporal dynamics of gene expression is vital for interpreting biological responses, especially in drug treatment studies. Conventional visualization techniques, such as heatmaps and static clustering, often fail to effectively capture these temporal dynamics, particularly when analyzing large-scale multidimensional datasets. These traditional methods tend to obscure fine-grained temporal transitions, resulting in overcrowded visualizations, diminished clarity, and limited interpretability of biologically significant patterns.

**Methods:**

To address these visualization challenges, we introduce Temporal GeneTerrain, an advanced method designed to represent dynamic changes in gene expression over time. We applied Temporal GeneTerrain to compare transcriptomic perturbations induced by mefloquine (M), tamoxifen (T), and withaferin A (W), both individually and in all-pairwise and triple combinations (TM, TW, MW, and TMW), in LNCaP prostate cancer cells using the GSE149428 dataset (0, 3, 6, 9, 12, and 24 h). Expression values were first Z-score normalized, and the 1,000 most variably expressed genes were selected. To ensure coordinated temporal dynamics, we calculated Pearson correlation coefficients among these genes and retained those with r ≥ 0.5, resulting in 999 strongly co-expressed candidates. We then constructed a protein-protein interaction network for these genes and embedded it in two dimensions using the Kamada-Kawai force-directed algorithm. Finally, for each time point and treatment, we mapped the normalized expression values of the corresponding genes onto the fixed Kamada-Kawai layout as Gaussian density fields (σ = 0.03), generating a distinct Temporal GeneTerrain map for each time-condition combination.

**Results:**

The application of Temporal GeneTerrain revealed intricate temporal shifts in gene expression, particularly unveiling delayed responses in pathways such as NGF-stimulated transcription and the unfolded protein response under combined drug treatments. Compared to traditional heatmap visualizations, Temporal GeneTerrain significantly improved both resolution and interpretability, effectively capturing gene expression patterns’ multidimensional and transient nature. This enhancement provides a solid foundation for further research and analysis, assuring the scientific community of the method’s reliability.

**Discussion:**

Temporal GeneTerrain addresses the limitations of traditional visualization methods by offering an intuitive and detailed representation of gene expression dynamics. Compared to other approaches, such as heatmaps and static clustering, Temporal GeneTerrain uniquely captures the transient nature of gene expression patterns. This method significantly enhances the interpretability of complex biological datasets, thereby supporting informed decision-making in biological research and therapeutic development.

## 1 Introduction

Precision medicine, at its core, centers on selecting optimal treatments for individual patients. Given the heterogeneity of patients’ genetic profiles, it is crucial to identify appropriate drugs and to administer them at the most effective time or disease stage. In translational science, extensive efforts have focused on characterizing gene function and determining the drugs specifically targeting those genes. Importantly, as a disease progresses, its molecular drivers--the specific genes or gene sets involved--may change, influencing both disease propagation and therapeutic outcomes. This dynamic nature necessitates treatment strategies that adapt over time, a challenge underscored by *in vitro* drug screening studies, where traditional visualization techniques such as heat maps, clustering, bar charts, and box plots, are employed to represent drug-induced gene expression changes (Friedman et al., 2015; McGranahan and Swanton, 2015; Huang et al., 2017; Konno et al., 2019; Malone et al., 2020; Zeng et al., 2021).

Traditional gene expression visualization techniques have been instrumental in depicting transcriptomic changes and discerning patterns for various conditions within complex datasets. Heat maps effectively capture expression gradients but cannot typically illustrate gene interactions or integrate functional annotations. Clustering methods, including self-organizing maps (Kohonen, 1997; Tamayo et al., 1999), can handle large-scale data, yet often yield results that are difficult to interpret or may not accurately reflect the underlying biology. Bar charts and box plots provide useful summaries but are limited in representing the complexity and multidimensionality of gene expression profiles (Tamayo et al., 1999).

Moreover, these conventional techniques suffer from data overcrowding and loss of resolution when applied to large datasets (Dietzsch et al., 2006; Katz et al., 2010; Deng et al., 2014; Metsalu and Vilo, 2015), and are inadequate for capturing dynamic interactions and temporal transitions in gene activity. Clustering methods may not always reflect the actual biological function of genes, leading to potential misinterpretations (Eisen et al., 1998; Tamayo et al., 1999). In addition, a general shortcoming of conventional visualization techniques is their limited integration of different data types that overlook potential correlations, trends, or causal relationships (Gehlenborg and Wong, 2012).

These limitations underscore the need for more advanced, flexible, and comprehensive visualization methods. Newer techniques are required to handle the complexity of gene expression data while providing interactive and integrative capabilities for deeper insights and interpretations. Such advanced methods could significantly enhance our understanding of gene interactions and functions, particularly in complex biological systems, and offer a more comprehensive view of drug responses in cancer cell lines over time.

While traditional visualization approaches effectively capture gene expression at discrete time points, they fail to convey the continuous evolution of gene regulatory networks during disease progression. The temporal aspect of gene involvement is critical; genes may be differentially engaged as the disease evolves, influencing both progression and treatment outcomes. Static representations do not capture these transitions, limiting our understanding of the dynamic interplay between gene regulation and therapeutic interventions.

In response to these challenges, we propose the Temporal GeneTerrain, a novel visualization technique designed to capture and represent dynamic changes in gene expression over time. Unlike static snapshots, Temporal GeneTerrains generate a continuous, integrated view of gene expression trajectories that evolves during disease progression and treatment response. By revealing temporal transitions in gene expression, this method provides new insights into the molecular dynamics underlying pathophysiological and therapeutic responses.

Temporal GeneTerrains build upon established visualization concepts while introducing several key innovations. Rather than displaying a series of disconnected time-point snapshots, our approach creates a continuous representation that emphasizes the trajectories of expression changes. This design facilitates the identification of coordinated expression patterns, transient effects, and delayed responses that might be overlooked using conventional methods. Furthermore, the technique incorporates interactive elements that enable users to explore the data at different levels of granularity, focusing on specific genes, pathways, or time periods of interest to gain multiscale insights (Ahmed et al., 2021).

A significant strength of Temporal GeneTerrains is their capacity to manage large-scale, multidimensional datasets without compromising interpretability. Dimensionality reduction algorithms preserve biologically meaningful relationships, allowing complex temporal patterns to be visualized intuitively. This capability is especially valuable in precision medicine, where integrating heterogeneous data sources is essential for informing clinical decisions.

Another distinctive feature of Temporal GeneTerrains is its integration of functional annotations and interaction networks within the visualization. By overlaying known biological pathways, protein-protein interactions, and gene-disease associations, the technique provides context for interpreting observed expression changes. This integrative approach bridges the gap between molecular data and clinical implications, facilitating the translation of research findings into actionable insights for patient care (Gonzalez-Hernandez et al., 2018).

Key innovations of Temporal GeneTerrain include:

• **Continuous Temporal Mapping**: Rather than discrete snapshots, our method interpolates expression changes to form a smooth trajectory, exposing transient waves and sustained shifts in gene activity.
• **Integration of Functional Context**: By overlaying pathway annotations and PPI connections, each terrain conveys mechanistic insights, linking molecular interactions to dynamic expression patterns.
• **Invariant Network Topology:** Re-optimizing layouts at each time point introduces visual jitter, impeding clear trend tracking. Freezing node coordinates on a single baseline layout eliminates variability, enabling unambiguous comparison of gene trajectories over time.
• **Adaptive Noise Smoothing:** Fixed smoothing can either blur sharp spikes or overemphasize noise. Dynamic modulation of the GeneTerrain’s parameter according to expression-change magnitude sharpens meaningful transients and highlights sustained patterns, balancing sensitivity and clarity.
• **Scalability and Interactivity**: Advanced dimensionality-reduction techniques ensure that even large, multidimensional datasets remain interpretable. Interactive controls allow users to explore different temporal resolutions and focus on specific subnetworks (Ahmed et al., 2021).

In Table 1, we compared Temporal GeneTerrain against complementary dynamic-network and trajectory-inference approaches. These include TS-OCD for detecting overlapping temporal protein complexes (Ou-Yang et al., 2014), DyNet/DyNetViewer for synchronized network timelines (Goenawan et al., 2016), TimeNexus for multilayer network construction (Pierrelée et al., 2020), TVNViewer for web-based rewiring exploration (Curtis et al., 2011), NACEP for module-aware expression comparison (Huang et al., 2010), TETRAMER for modeling temporal transcriptional-regulation cascades (Cholley et al., 2018), and BioTapestry for hierarchical regulatory maps (Longabaugh, 2011). TSEE excels at uncovering latent trajectories in single-cell landscapes by embedding temporal information directly into a dimensionality-reduction framework (An et al., 2019). TrendCatcher provides a robust statistical toolkit for pinpointing and visualizing distinct gene- and pathway-level dynamics from longitudinal data (Wang et al., 2022). While each of these tools represents a state-of-the-art solution within its specialized domain, Temporal GeneTerrain complements them by fusing temporal dynamics with molecular interaction networks into an interpretable spatial terrain, making it uniquely suited for studies where understanding the co-regulation and modular behavior of genes over time is critical. Supplementary 1 provides additional details on the implementation and capabilities of each method.

**TABLE 1:** Feature matrix comparing Temporal GeneTerrain against leading dynamic gene-expression visualization tools.

Method	Continuous temporal map	PPI integration (spatial)	Statistical DDEG modeling	Regulatory GRN modeling	Cytoscape Plugin
Temporal GeneTerrain	✔	✔	✖	✖	✖
TSEE	✔	✖	✖	✖	✖
TrendCatcher	✖	✖	✔	✖	✖
TS-OCD	✖	✔	✖	✖	✖
DyNet	✖	✔	✖	✖	✔
DyNetViewer	✖	✖	✖	✖	✔
TimeNexus	✖	✖	✖	✖	✔
TVNViewer	✖	✖	✖	✖	✔
NACEP	✖	✖	✔	✖	✖
TETRAMER	✖	✖	✖	✔	✖
BioTapestry	✖	✖	✖	✔	✖

✔ indicates that the method provides the listed capability; ✖ denotes lack thereof.

As a proof of concept, we applied Temporal GeneTerrains to study the effects of single drug perturbations and their combinations in prostate cancer cell lines. Prostate cancer was chosen as an ideal model due to its well-characterized progression patterns and variability in patients’ treatment responses. Using our generated temporally integrated GeneTerrain visualizations, we observed distinct gene expression transitions throughout treatment, revealing both immediate and delayed responses that static methods failed to capture.

Subsequent gene set enrichment analysis further enhanced the biological interpretation of our results by associating coordinated gene expression changes with key pathways implicated in disease progression and drug sensitivity or resistance. These insights underscore the potential of Temporal GeneTerrains to elucidate molecular processes that drive disease dynamics, and to identify novel targets for therapeutic intervention (Zhang et al., 2022).

The remainder of the paper is structured as follows: Section 2 details the datasets involved and the algorithmic design of the Temporal GeneTerrain method. Section 3 presents a comprehensive evaluation of results from a prostate cancer cell line case study under various drug perturbations. Section 4 discusses the biological significance, clinical implications, and potential applications of our findings, concluding with a summary of contributions and suggestions for future research.

## 2 Materials and methods

### 2.1 Dataset

The GSE149428 dataset (Diaz et al., 2020) was retrieved from the Gene Expression Omnibus (GEO) Database (https://www.ncbi.nlm.nih.gov/geo/query/acc.cgi?acc=GSE149428). This dataset comprises transcriptomic profiles of the LNCaP prostate cancer cell line treated with DMSO (vehicle control), three distinct drugs, and all possible combinations of these drugs, with each condition performed in triplicate. Samples were collected at six time intervals (0, 3, 6, 9, 12, and 24 h), including a triple drug combination explicitly evaluated in LNCaP cells.

### 2.2 Temporal GeneTerrain: Conceptual framework and implementation

The Temporal GeneTerrain consists of an innovative visualization methodology that extends conventional gene expression analysis by incorporating temporal dynamics into a network-spatial framework that enables researchers to observe the evolution of gene expression patterns over time within a biologically meaningful spatial context. The method builds upon the GeneTerrain visualization framework initially proposed by You et al. (2010) by incorporating an additional temporal dimension that reveals dynamic changes in gene expression patterns. The implementation follows a two-phase process: (1) generating individual GeneTerrain visualizations at discrete time points and (2) temporally integrating these visualizations to reveal continuous dynamic expression patterns.

### 2.3 Data preparation and preprocessing

Before visualization, gene expression data were normalized using Z-score normalization to standardize measurements across time points and conditions. Differential expression analysis was performed at each time point relative to baseline or control samples. Protein-protein interaction (PPI) network data were obtained from HAPPI (Chen et al., 2017) and BEERE (Yue et al., 2019) to provide the underlying network structure for network-based visualization.

### 2.4 GeneTerrain generation

#### 2.4.1 Network layout construction

Each GeneTerrain visualization is founded on a two-dimensional spatial layout, where each node represents a gene, and the inter-node distances reflect functional relationships derived from the PPI network. To construct this layout, we employed the Kamada-Kawai force-directed graph drawing algorithm (Kamada and Kawai, 1989) to generate this layout based on protein-protein interaction data. This algorithm positions genes in two-dimensional space such that genes with functional relationships (as defined by the PPI network) are placed in closer proximity.

The Kamada-Kawai algorithm (Kamada and Kawai, 1989) determines optimal vertex (gene) positioning by minimizing the system’s energy, which is computed using a combination of attractive forces between connected vertices and repulsive forces between all pairs of vertices. The system energy (E) is formulated as:

$$E=\sum_{\left(i,j\right)\in E}\frac{k^{2}{\left(d_{ij}-l_{ij}\right)}^{2}}{l_{ij}}+\sum_{i\neq j}\frac{k^{2}}{d_{ij}}$$

Where:

• E represents the set of edges in the PPI network
• $d_{ij}$ is the Euclidean distance between nodes *i* and *j* in the visualization
• $l_{ij}$ represents the desired distance between nodes *i* and *j*, computed as:

$$l_{ij}=k\sqrt{\frac{Area}{\left|V\right|}\;}\;L_{ij}$$

• |V| is the total number of vertices (genes) in the graph
• Area is the display area of the visualization
• $L_{ij}$ is the shortest path length between nodes *i* and *j* in the PPI network
• k is a constant scaling factor

The algorithm iteratively adjusts vertex positions to minimize this energy function until convergence criteria are met.

### 2.5 Gene expression signal visualization

After constructing the network layout, gene expression data are mapped onto this spatial framework by generating a continuous signal field. Each gene’s expression level is modeled as a three-dimensional Gaussian distribution centered at its corresponding coordinates in the layout. The amplitude of the Gaussian reflects the gene’s expression value, where positive amplitudes denote upregulation and negative amplitudes denote downregulation.

The continuous signal S (*x,y*) at any point (*x,y*) in the visualization is given by:

$$S\left(x,y\right)=\sum_{i=1}^{n}e_{i}\cdot \exp\left(-\frac{{\left(x-x_{i}\right)}^{2}+{\left(y-y_{i}\right)}^{2}}{2\sigma^{2}}\right)$$

Where:

• $e_{i}$ is the expression value of gene i

• ( $x_{i}$ , $y_{i}$ ) are the spatial coordinates of gene i in the layout
• σ controls the dispersion (width) of the Gaussian distribution

The resulting continuous signal field is rendered using a divergent color scheme, with intense red indicating strong upregulation, intense blue indicating strong downregulation, and intermediate values represented by shades of green. Figure 1 of Supplementary 2 presents the complete GeneTerrain workflow and demonstrates how varying the Gaussian smoothing width (σ) systematically modulates the spatial resolution and feature sharpness of the resulting terrain.

**FIGURE 1:**
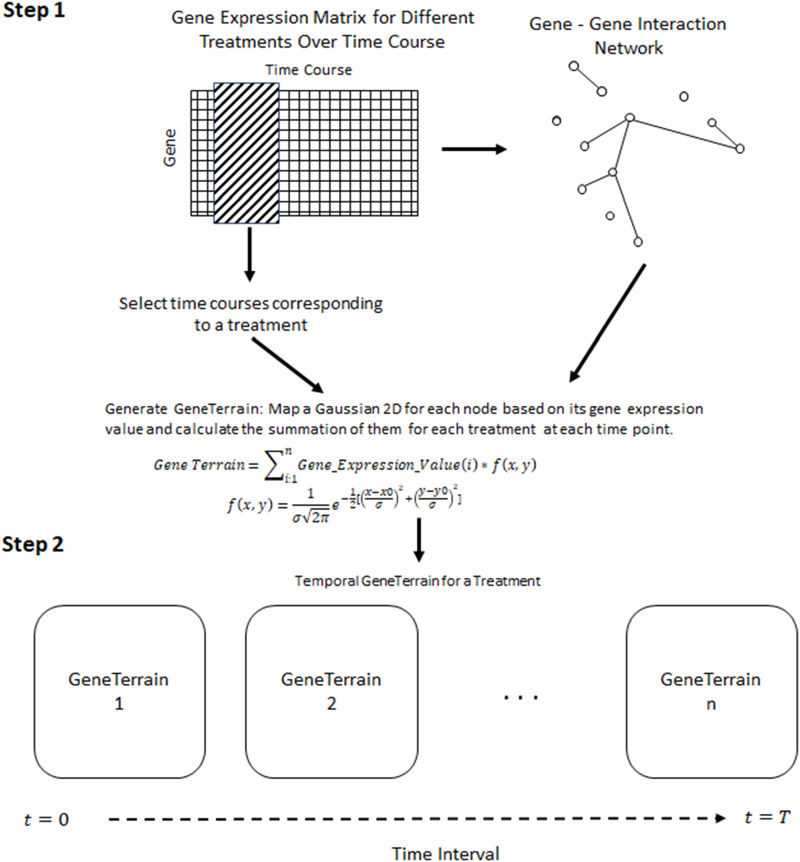
A framework for constructing Temporal GeneTerrains involves Step-1, Generating the GeneTerrain for visualizing using the network biology of genes and gene signals; Step-2, Stitching the GeneTerrain generated in Step-1 based on their chronological order.

### 2.6 Temporal integration

The second phase of the Temporal GeneTerrain method involves integrating individual visualizations to capture temporal dynamics. For each cell line, a series of GeneTerrain visualizations was generated for discrete time points (e.g., 0, 6, 12, 24, and 48 h). To facilitate direct comparison across time, the network layout coordinates were held constant while updating the gene expression values for each time point. This strategy enables the clear visualization of temporal trajectories in gene expression, thereby revealing coordinated regulatory changes and identifying genes with significant temporal modulation. Figure 1 schematically illustrates the framework for constructing and temporally integrating GeneTerrains, and Algorithm 1 presents the pseudocode for the proposed method.

Algorithm 1 Pseudocode of Temporal GeneTerrain Method.

**Require:** GeneExpressionData, PPINetwork, TimePoints, *σ*, *k*, area, *ϵ*, max- Iterations, *η*
**Ensure:** TemporalVisualization
1: **Data Preprocessing:**
2: **for** each sample in GeneExpressionData **do**
3:  Normalize (e.g., via Z-score)
4: **end for**
5: **Network Layout Construction:**
6: Let V=set of genes in PPINetwork
7: **for** each pair $\left(i,j\right)\in V,i\neq j$ **do**
8: $L_{ij}\;\leftarrow \text{ ShortestPath}\left(\text{PPINetwork},i,j\right)$

9: $l_{ij}\;\leftarrow \;k\sqrt{\frac{area}{\left|V\right|}}L_{ij}$

10: **end for**
11: **for** each gene i∈V **do**
12:  Initialize position $p_{i}$

13: **end for**
14: $iteration\;\leftarrow \;0,E_{\text{prev}}\;\leftarrow \infty$

15: **while** iteration < maxIterations **do**
16: $E_{\text{cur}}\leftarrow 0$

17:  **for all** i,j∈V,i≠j **do**
18: $d_{ij}\leftarrow \left\|p_{i}-p_{j}\right\|$

19: $E_{ij}\leftarrow \frac{k^{2}{\left(d_{ij}-l_{ij}\right)}^{2}}{l_{ij}}+\frac{k^{2}}{d_{ij}}$

20: $E_{cur}\leftarrow E_{cur}+E_{ij}$

21:  **end for**
22:  **if** $\left|E_{\text{prev}}-E_{\text{cur}}\right|<\;\epsilon$ **then**
23:   **break**
24:  **end if**
25:  **for** each i∈V **do**
26: $G_{i}\leftarrow \sum_{j\neq i}\nabla_{p_{i}}E_{ij}$

27: $p_{i}\leftarrow p_{i}-\eta \;G_{i}$

28:  **end for**
29: $E_{\text{prev}}\;\leftarrow \;E_{\text{cur}},iteration\;\leftarrow \;iteration+1$

30: **end while**
31: **Signal Field Generation:**
32: **for** each t∈TimePoints **do**
33:  **for** each grid point (*x, y*) **do**
34: $S_{t}\left(x,y\right)\leftarrow \sum_{i\in V}e_{i}\left(t\right)\exp\left(-\frac{{\left(x-p_{i,x}\right)}^{2}+{\left(y-p_{i,y}\right)}^{2}}{{2\sigma }^{2}}\right)$

35:  **end for**
36:  Map $S_{t}\left(x,y\right)$ to a color map $V_{t}$

37: **end for**
38: Temporal Integration:
39: Assemble $\left\{V_{t}\right\}$ into TemporalVisualization
40: **return** TemporalVisualization

## 3 Results

In this article, we employed the Temporal GeneTerrain methodology to perform a comparative analysis of transcriptomic perturbations induced by three drugs, mefloquine (M), tamoxifen (T), and withaferin A (W), administered individually and in combination (TM, TW, MW, and TMW) on the LNCaP prostate cancer cell line. This case study demonstrates how Temporal GeneTerrain can elucidate both single-drug and combination-based perturbation at the transcriptomic level. We used the GSE149428 dataset (Diaz et al., 2020) for our case study, which consists of the drug perturbation data from cancer cell lines cultured for six distinct time intervals (0, 3, 6, 9, 12, and 24 h).

**FIGURE 2:**
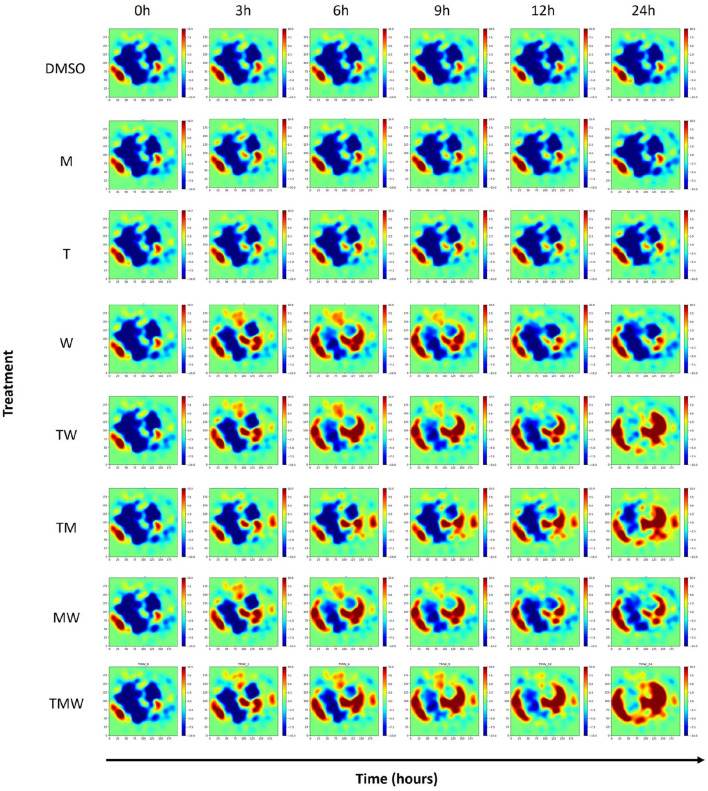
GeneTerrains at different time intervals forming the Temporal GeneTerrain for DMSO as the control vehicle, three treatments, and their combination for the LNCaP cancer cell line.

We initially selected 1,000 genes exhibiting high expression variance for data preprocessing, a key indicator of dynamic regulation and potential interaction with drug response. To refine our gene set, we conducted a thorough correlation analysis to identify genes with a correlation coefficient threshold above 0.5. This stringent selection criterion allowed us to distill our initial group to a core subset of 999 genes, demonstrating a strong inter-correlation, suggesting a significant role in drug sensitivity and resistance mechanisms. Parameter optimization involved manually setting the sigma parameter for the GeneTerrain algorithm to 0.03. Subsequently, GeneTerrain visualizations were generated for all samples. Figure 2 illustrates GeneTerrain visualizations that capture the dynamics of gene expression alterations across successive time intervals (0→3, 3→6, 6→9, 9→12, and 12→24 h). At each interval, the GeneTerrains identify sets of significantly upregulated or downregulated genes; these gene sets are subsequently subjected to enrichment analysis to elucidate critical pathways underpinning the observed temporal dynamics. The top row corresponds to the DMSO (vehicle control), while the following three rows display results from single-drug treatments, and the final four rows depict drug combination treatments. Notably, treatments M and T exhibit minimal perturbations, maintaining relatively consistent expression patterns over the 24-h period. In contrast, other remaining treatments display pronounced gene expression alterations. The color-coded patterns and trajectories depicted in the GeneTerrain maps effectively capture these dynamic perturbations over time, with a colormap range from −10 to +10.

**FIGURE 3:**
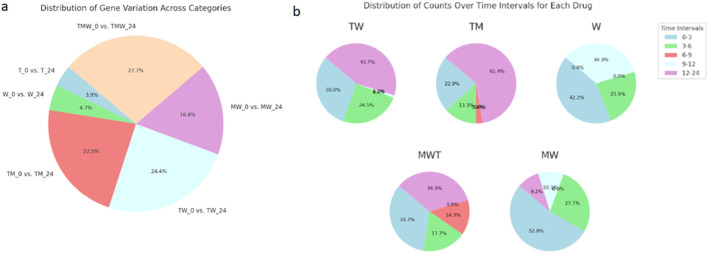
**(a)** a pie chart representing the distribution of gene variation across specified categories: T_0 vs. T_24, W_0 vs. W_24, TM_0 vs. TM_24, TW_0 vs. TW_24, MW_0 vs. MW_24, and TMW_0 vs. TMW_24. Each segment’s size corresponds to the proportion of genes with high variation in that category, and the percentages are shown for each segment. **(b)** The pie chart shows the percentage of each portion, representing the distribution of counts over the various time intervals for each drug (MWT, MW, TW, TM, W) in the time intervals (0–3, 3–6, 6–9, 9–12, 12–24 h).

Figure 3 provides an in-depth portrayal of gene expression variability in prostate cancer cells subjected to different drug treatments across time, organized into two key sections. Figure 3a features a pie chart detailing gene variation distributions across six comparison groups: T_0 vs. T_24, W_0 vs. W_24, TM_0 vs. TM_24, TW_0 vs. TW_24, MW_0 vs. MW_24, and TMW_0 vs. TMW_24. Each chart segment corresponds to one comparison category, with segment sizes reflecting the proportion of highly variable genes in each. This format clarifies how each treatment comparison uniquely influences gene expression variability, accompanied by percentage values for quantitative insight. The TMW treatment displays the most significant variability between its initial and 24-h time points. Panel 3b highlights temporal patterns of gene expression changes by presenting a pie chart that illustrates the percentages of gene variability across discrete time intervals (0–3, 3–6, 6–9, 9–12, and 12–24 h) for individual drug treatments (TMW, MW, TW, TM, W). The segments, representing distinct time intervals, offer insights into the dynamic nature of cellular responses, precisely pinpointing substantial changes during the TM treatment at 12–24 h, the MW treatment at 0–3 h, and the TMW treatment at 12–24 h. Notably, the minimal gene expression changes observed in the M and T treatments, encompassing both upregulated and downregulated genes, were omitted from Figure 3.

**FIGURE 4:**
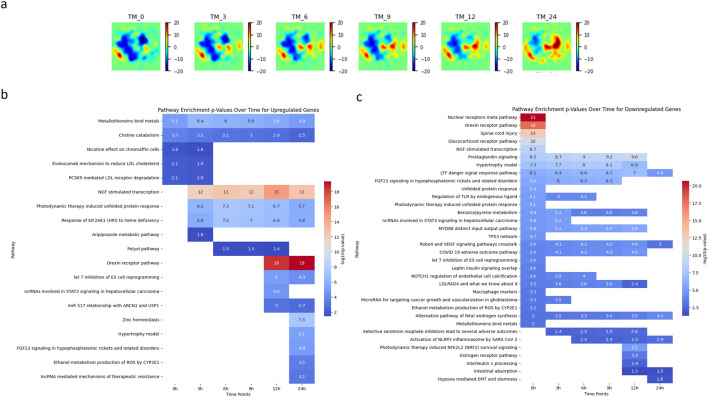
Analysis of Pathway Dynamics in LNCaP Cancer Cell Line Under TM Treatment. **(a)** illustrating the Temporal GeneTerrain of a LNCaP cancer cell line sample treated with a combination TM treatment, cultured over intervals of 0, 3, 6, 9, 12, and 24 h. **(b, c)** Two heatmaps of visualizing the synthetic pathway enrichment scores over time for TM treatment for genes that are upregulated and downregulated using the PAGER tool.

Figure 4a presents the Temporal GeneTerrain visualization for the TM treatment, depicting drug perturbations on a cancer cell line sample cultured over intervals of 0, 3, 6, 9, 12, and 24 h. Figures 4b, c present heatmaps of synthetic pathway enrichment scores over time in the TM treatment study, utilizing the PAGER tool (Yue et al., 2018; Yue et al., 2015) for both upregulated and downregulated gene sets. Each heatmap displays the enrichment level for various pathways at different time points, with rows representing pathways and columns denoting time points. Each cell’s color intensity and values reflect the pathway’s enrichment score. The PAGER tool parameters were set to a PAG range of 2–5,000, a similarity score threshold of 0.05, a minimum overlap of one gene, a cohesion value of 1, and a p-value threshold of 0.05, utilizing data from the WikiPathways_2021 dataset for *Homo sapiens*. The heatmaps represent p-values on a–log10 scale to enhance the visualization of statistical significance, with more intense red shading indicating higher pathway significance.

Examination of the pathway heatmap for upregulated genes in Figure 4b provides a nuanced view of the cellular response dynamics in the LNCaP cell line over the 24-h period (Horoszewicz et al., 1983). At 0 h, the metallothionein-binding pathway is notably active, with an expression level of 5.1, suggesting an immediate cellular response to the drug. This pathway remains consistently upregulated throughout the 24-h period, although with a gradual decrease in expression, indicating a sustained but diminishing response. The choline catabolism pathway shows a decrease in activity over time, starting at a moderate level at 0 h and progressively diminishing, reflecting a possible adaptation or downregulation of this pathway in response to the treatment. Notably, several pathways show no initial activity at 0 h but become significantly upregulated later. For instance, the NGF-stimulated transcription pathway (Chen et al., 2021) remains inactive at 0 h but shows a dramatic increase from 3 h onwards, peaking at 14.7 at 12 h. This suggests a delayed but robust response to the drug. Similarly, the photodynamic therapy-induced unfolded protein response (Firczuk et al., 2013) and the response of EIF2AK1 (HRI) (Cordova et al., 2022) to heme deficiency pathways are activated from 3 h onwards, indicating their roles in later stages of the drug response. Interestingly, pathways such as the orexin receptor pathway (Graybill and Weissig, 2017) and zinc homeostasis (Li et al., 2020) remain inactive until later stages (12 and 24 h, respectively) before exhibiting markedly high expression levels, suggesting involvement in long-term cellular adaptations. The heatmap in Figure 4c reveals distinct temporal response patterns among various pathways in the LNCaP cancer cell line across the time points of 0, 3, 6, 9, 12, and 24 h. At 0 h, pathways, including the nuclear receptors’ meta pathway, the orexin receptor pathway, and spinal cord injury-related pathways, exhibit significant activation, suggesting an immediate cellular response to the drug perturbation. However, these pathways show no activity at subsequent time points, suggesting a transient or initial phase response. Subsequently, other pathways become active as time progresses. Notably, the prostaglandin signaling pathway shows a consistent increase in activity from 3 to 12 h, indicating a sustained response to the drug over this period. Similarly, the LTF danger signal response pathway exhibits a gradual increase from 3 to 24 h, with a slight dip at the 12-h mark, suggesting a prolonged involvement in the drug response. In contrast, some pathways, such as the FGF23 signaling and unfolded protein response, show activity only in the initial hours (up to 6 h) and then cease to respond, implying a role in early-stage drug response mechanisms. Interestingly, pathways associated with selective serotonin reuptake inhibitors and activation of the NLRP3 inflammasome by SARS-CoV-2 exhibit delayed activation, with no initial activity but subsequent triggering at 3 and 6 h, further highlighting temporal specificity in drug response.

These pathways’ temporal specificity and high significance suggest distinct roles in mediating the cellular response to drug treatment. These findings are instrumental in identifying potential targets for cancer therapy, elucidating the timing of cellular responses, and informing the design of time-dependent therapeutic interventions. Overall, the temporal pathway activation patterns in LNCaP cells reveal phase-specific biological responses to treatment. Nuclear receptor meta-pathway activation at baseline (0 h) aligns with studies showing elevated LRH-1 expression in castration-resistant prostate cancer (CRPC), where this nuclear receptor drives *de novo* androgen biosynthesis via steroidogenic enzymes (*CYP17A1*, *HSD3B1*) (Xiao et al., 2018). The transient orexin receptor pathway activity at 0 h corresponds to OX1R’s dual role in prostate cancer - baseline receptor presence in LNCaP cells (Graybill and Weissig, 2017) with therapeutic potential through androgen receptor translocation inhibition (Graybill and Weissig, 2017; Couvineau et al., 2021). However, its rapid deactivation likely reflects receptor internalization or downstream signaling adaptation. The sustained activation of prostaglandin signaling from 3 to 12 h may intersect with NF-κB-mediated survival pathways, as neuropeptides such as bombesin have been shown to activate NF-κB in LNCaP cells to preserve androgen receptor (AR) stability under castration conditions (Jin et al., 2008). Similarly, the prolonged activation of the LTF danger signal mirrors mechanisms observed in CRPC progression, where chronic NF-κB activation prevents tumor regression through AR/cyclin D1 maintenance (Jin et al., 2008). The delayed NLRP3 inflammasome activation (from 6 h) may reflect secondary stress responses, potentially linked to LRH-1-mediated steroidogenesis creating pro-inflammatory microenvironments (Xiao et al., 2018). Although FGF23 signaling was not directly examined in these studies, its early-phase activity may be attributable to growth factor crosstalk during the initial response to treatment.

## 4 Discussion

This study presents Temporal GeneTerrain, an innovative visualization methodology that significantly enhances our ability to interpret the dynamic landscape of gene expression in response to pharmacological interventions. Moving beyond conventional static approaches such as heat maps and standard clustering techniques, this method effectively captures the temporal evolution of gene expression with enhanced resolution and biological relevance. Similar advancements have been observed in tools like TrendCatcher, which identifies dynamic transcriptional signatures and biological processes over time, further validating the importance of temporal analysis in transcriptomics (Wang et al., 2022).

Our analysis revealed specific dynamic transcriptional events in LNCaP cells, namely, the transient NGF-stimulated transcription surge peaking at 12 h, the short-lived stress-response module activation between 6 and 9 h, the delayed zinc homeostasis upregulation at 24 h, and the early but transient orexin receptor pathway engagement at baseline. Previous studies examining NGF effects in LNCaP cells focused primarily on proliferation assays conducted over multi-day intervals, without accompanying genome-wide expression profiling at intermediate time points (Sortino et al., 2000). Earlier transcriptomic analyses characterizing LNCaP progression toward castration resistance sampled only broad intervals (e.g., 0, 3, 6, 12, and 24 h), without highlighting transient activation spikes (Vaarala et al., 2000). Investigations of time-course zinc treatments in prostate cancer cells predominantly profiled gene expression at earlier stages (e.g., 3 and 6 h) and did not capture the late-phase activation at 24 h (Lin et al., 2009; Kolenko et al., 2013). Similarly, studies of orexin receptors reported negligible OX1R expression in standard LNCaP cells and lacked temporally resolved gene expression data that could reveal an immediate baseline engagement (Saghapour et al., 2024). Furthermore, while methods such as TrendCatcher have demonstrated the utility of fine-grained temporal analyses in other contexts, such as COVID-19 PBMC transcriptomes (Wang et al., 2022), and tools like TSEE have underscored the importance of high-resolution temporal embedding in single-cell RNA-seq data (An et al., 2019), analogous approaches have not previously been applied to the GSE149428 LNCaP dataset.

This echoes findings from studies like the GeneTerrain Knowledge Map (GTKM), which uses protein-protein interaction networks to graphically represent differentially expressed genes, offering nuanced insights into gene interactions and expression patterns (Saghapour et al., 2024). Notably, Temporal GeneTerrain adeptly delineates immediate and delayed transcriptional responses to single and combination treatments with mefloquine, tamoxifen, and withaferin A. This temporal dissection reveals that pathway activations, such as NGF-stimulated transcription and the unfolded protein response, occur in a staggered manner, aligning with studies that emphasize transcriptional adaptation as a key driver of tumor progression rather than a reflection of pre-existing cellular states (Bolis et al., 2021).

A principal advantage of the Temporal GeneTerrain framework is its integration of protein-protein interaction networks via force-directed layout algorithms. This approach provides a spatial representation that mirrors the functional interrelationships among genes, facilitating a more intuitive interpretation of underlying biological processes. By superimposing gene expression values on these networks using a continuous signal field, nuanced but significant shifts in cellular behavior over time were visualized. Similar approaches, such as TSEE (Time Series Elastic Embedding), have demonstrated the potential of integrating temporal information into visualization frameworks to enhance the resolution of dynamic transitions (An et al., 2019). The observed temporal dynamics suggest that while some genes respond promptly to treatment, others display delayed regulation, indicating a complex interaction between immediate drug effects and subsequent adaptive responses.

Furthermore, the integration of gene set enrichment analysis reinforces our visualization strategy. By mapping expression changes to known biological pathways, we confirm that the temporal shifts captured by Temporal GeneTerrain correspond to critical regulatory mechanisms. For instance, the delayed yet pronounced activation of pathways such as NGF-stimulated transcription underscores the prospective relevance of temporal regulation in influencing treatment outcomes. These findings are consistent with studies showing gradual transcriptional progression during prostate cancer adaptation to androgen deprivation therapy (Vaarala et al., 2000; Romanuik et al., 2010). This dual strategy—merging spatial network visualization with pathway enrichment analysis provides a comprehensive framework for interpreting high-dimensional transcriptomic data in clinically relevant contexts.

Unlike generating independent GeneTerrain snapshots for each time point, which requires realignment of hundreds of gene nodes, our temporal stitching strategy constructs a single force-directed layout at baseline and preserves those exact coordinates across all subsequent time points. By mapping time-specific expression fields onto an invariant topology and then interpolating between them, users can directly track the ‘flow’ of each gene’s activity without the visual jitter introduced by re-optimizing the layout at every interval.

To support this, we modified the underlying algorithm in two ways. First, we performed Kamada–Kawai embedding just once on the PPI network and froze the node positions for all time points, which eliminates layout variability. Second, we introduced an adaptive Gaussian smoothing scheme, in which σ is automatically adjusted based on the magnitude of expression change between consecutive intervals such that smaller σ values sharpen transient spikes, while larger σ values emphasize sustained trends. Together, these enhancements sharpen the contrast between immediate versus delayed responses and reduce noise.

Despite these promising findings, several limitations merit consideration. First, the accuracy of the spatial layouts produced by our methodology is inherently dependent on the quality and completeness of the underlying protein-protein interaction data. Incomplete or inaccurate interaction networks could obscure subtle gene relationships. In addition, the parameter selection process, such as determining the optimal sigma value for Gaussian smoothing, requires careful calibration. Although our settings were informed by preliminary analyses, further optimization across diverse datasets is necessary to establish standardized protocols, a challenge similarly encountered in other time-series visualization frameworks like TSEE (An et al., 2019).

Despite the advances afforded by Temporal GeneTerrain, our approach is inherently dependent on the quality and completeness of the underlying protein–protein interaction (PPI) network. Gaps or inaccuracies in PPI data can obscure true functional relationships and lead to misplacement of genes in the terrain. In principle, our framework can accommodate alternative network priors such as regulatory interactions derived from ChIP-seq, RIP-seq, or Hi-C chromatin-contact maps by simply substituting the PPI adjacency matrix used in the force-directed layout. Employing a ChIP-seq–based network, for example, would emphasize direct transcription factor–target relationships, whereas Hi-C–derived contacts could reveal three-dimensional co-regulation modules. Similarly, our high-variance gene filter was chosen to focus on the most dynamically regulated genes, but more stringent criteria (e.g., top 500 most variable) would produce a sparser terrain with reduced visual clutter at the expense of potentially missing subtler but biologically important signals, while looser thresholds (e.g., top 2,000) would increase coverage but could overwhelm the visualization with noise. Systematic evaluation of these tradeoffs across multiple datasets would be essential for establishing best practices for gene selection.

Scalability to larger cohorts and more heterogeneous clinical samples also presents challenges. While we demonstrated Temporal GeneTerrain on ∼1,000 highly variable genes in a controlled cell-line experiment, extending this method to complete transcriptomes or patient-derived multi-omics (e.g., scRNA-seq, proteomics, metabolomics) would require strategies to manage both computational load and visual interpretability. Possible solutions include hierarchical terrain generation first mapping modules or pathway-level aggregates before “drilling down” to individual genes and GPU-accelerated force-directed layouts to handle millions of interactions. For clinical datasets, batch effects and sample heterogeneity can distort both the network and the temporal signal; incorporation of robust normalization, batch-effect correction, and adaptive σ-smoothing schemes will be necessary to ensure that inter-patient variability does not confound the temporal trajectories. Future work will focus on integrating these strategies to bring Temporal GeneTerrain toward large-scale, clinically actionable analyses.

As a result, Temporal GeneTerrains reveal biologically meaningful dynamics that discrete analyses would miss, such as the 3 h activation of NGF-stimulated transcription preceding any visible change in the unfolded protein response, and the transient peak in stress-response genes between 6 and 9 h that disappears by 12 h. These patterns emerge organically from the continuous terrain rather than as isolated signals across separate plots, significantly improving interpretability in time-series transcriptomic studies. When we used a traditional clustered heatmap to visualize the 0–24 h expression data, transient spikes were blurred and gene trajectories became misaligned. Figure 2 in Supplementary 2 shows how static heatmaps obscure fine-grained temporal transitions, with staggered clusters and shifted patterns. In contrast, our dynamic Temporal GeneTerrain, when using σ-modulated smoothing uncovers interaction patterns and transient activation waves, fully resolved underlying temporal dynamics.

Moreover, while Temporal GeneTerrains prove effective in visualizing dynamic gene expression in controlled *in vitro* environments, their application to more heterogeneous clinical samples may pose challenges. The variability inherent in patient-derived data encompassing differences in sample quality, treatment regimens, and disease stages could complicate the visualization and interpretation of temporal patterns. Future research should focus on adapting this methodology for clinical contexts, ideally integrating additional omics layers (e.g., proteomics or metabolomics) to yield a more holistic view of disease dynamics (Romanuik et al., 2010; Aviñó-Esteban et al., 2025).

Looking ahead, several promising avenues for future research emerge. Expanding the Temporal GeneTerrain framework to incorporate multi-omics data could provide a more enriched, systems-level perspective on cellular responses. Furthermore, coupling real-time data acquisition with interactive visualization tools may facilitate dynamic decision support in clinical settings, ultimately refining therapeutic precision. Such advancements would broaden our approach’s applicability and help bridge the gap between fundamental research and clinical practice.

In conclusion, Temporal GeneTerrains represent a significant methodological advancement in bioinformatics and precision medicine. By capturing the dynamic nature of gene expression and linking these alterations to functional biological pathways, this methodology provides researchers and clinicians with a powerful tool for understanding the complex temporal responses that underlie drug efficacy and disease progression. Continued enhancements and validation across diverse biological contexts will establish Temporal GeneTerrains as an indispensable resource for developing time-sensitive, personalized therapeutic strategies.

## Data Availability

The dataset utilized in this study can be found at: www.ncbi.nlm.nih.gov/geo/query/acc.cgi?acc=GSE149428, and the code is available at: https://github.com/aimed-lab/Temporal_GeneTerrain.
