# Supplementary material for: Temporal GeneTerrain: advancing precision medicine through dynamic gene expression visualization
Source: Front Bioinform. 2025 Jun 18;5:1602850. doi: 10.3389/fbinf.2025.1602850 (PMC12213653; doi:10.3389/fbinf.2025.1602850)
Supplement: Supplementary file 2 [file Presentation1.pptx]

## Slide 1
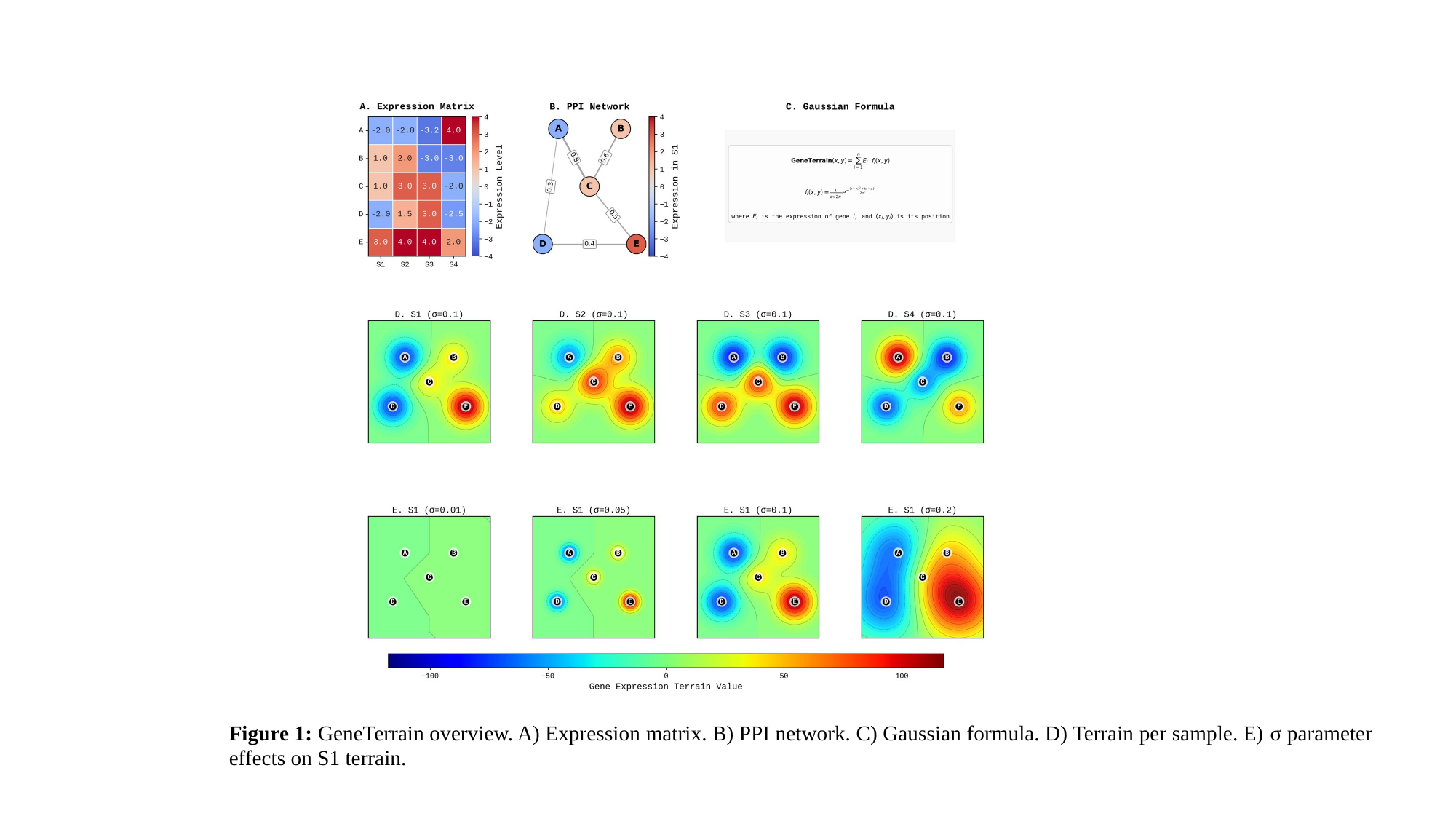

Figure 1: GeneTerrain overview. A) Expression matrix. B) PPI network. C) Gaussian formula. D) Terrain per sample. E) σ parameter effects on S1 terrain.

## Slide 2
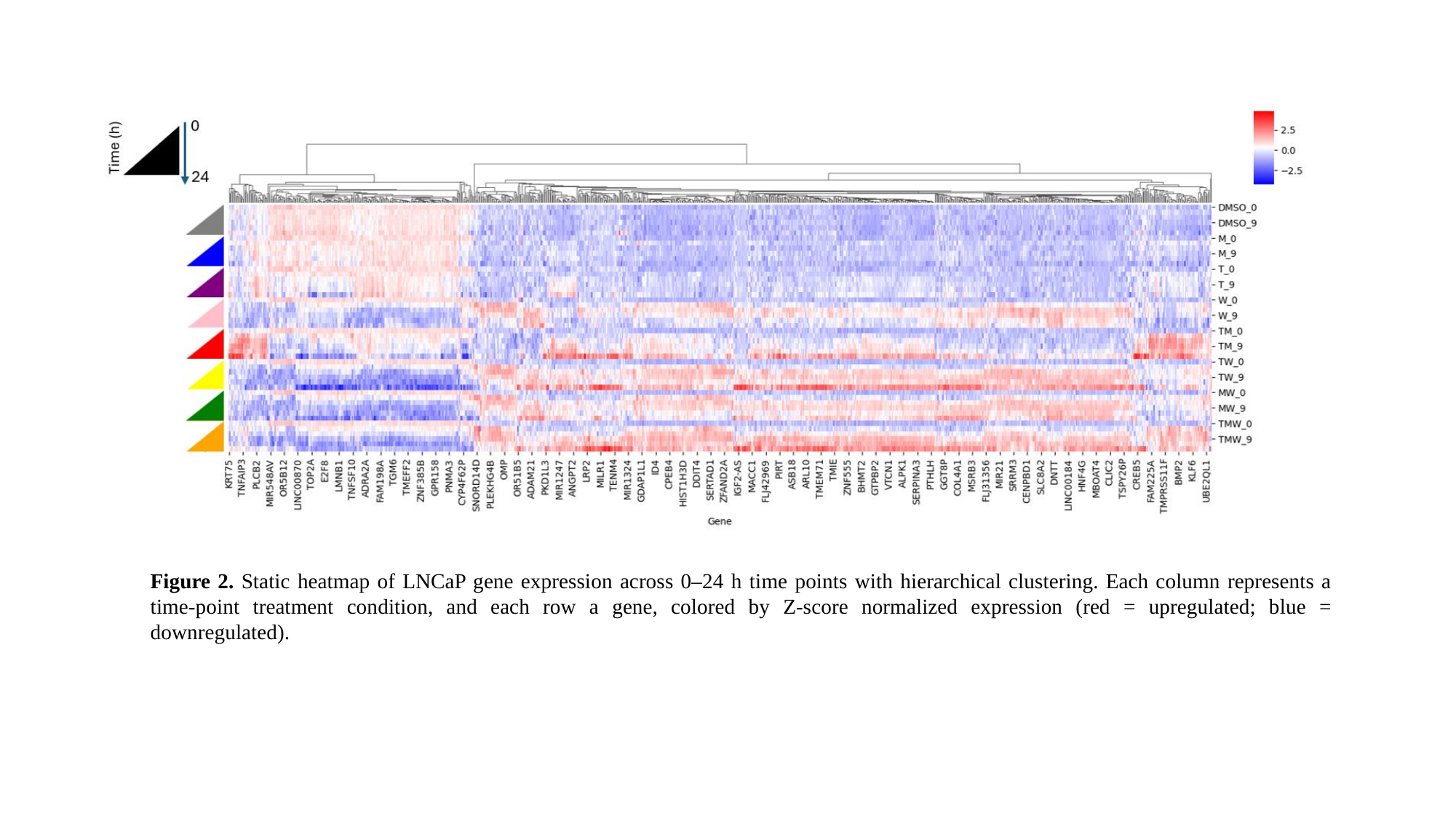

Figure 2. Static heatmap of LNCaP gene expression across 0–24 h time points with hierarchical clustering. Each column represents a time-point treatment condition, and each row a gene, colored by Z-score normalized expression (red = upregulated; blue = downregulated).
